# Hydralazine-Induced Vasculitis

**DOI:** 10.7759/cureus.35306

**Published:** 2023-02-22

**Authors:** Pulkit Gandhi, Bani Khurana, Ripudaman S Munjal, Arjun Sekar, Roopali Goyal Gandhi

**Affiliations:** 1 Nephrology, Rochester Regional Health, Rochester, USA; 2 Nutrition, Cornell University, Ithaca, USA; 3 Nephrology, St. Joseph Medical Center, Stockton, USA; 4 Internal Medicine, Rochester Regional Health, Rochester, USA

**Keywords:** anca-associated vasculitis, drug-induced acute kidney injury, immunosuppression, rituximab therapy, hydralazine-induced vasculitis

## Abstract

Hydralazine is a commonly prescribed medication which is used in the treatment of hypertension. While it is generally considered to be a safe and effective treatment, in rare cases it can cause a serious side effect known as hydralazine-induced vasculitis. Here we discuss this rare presentation in the form of a case report in a 67-year-old female with a past medical history of chronic obstructive pulmonary disease (COPD), congestive heart failure, hypertension, hyperlipidemia, left renal artery stenosis status post stenting who presented in the nephrology office for evaluation of recent worsening kidney function, and on further evaluation was found to have hematuria and proteinuria in the urine analysis. On further workup, she was noted to have severely elevated myeloperoxidase-antineutrophil cytoplasmic antibody (MPO-ANCA) titers with renal biopsy revealed very focal crescentic glomerulonephritis, an increased number of occlusive red blood cell cast with acute tubular necrosis. Mild interstitial fibrosis of <20% was seen and a diagnosis of drug-induced vasculitis from hydralazine was made.

## Introduction

Antineutrophil cytoplasmic antibody (ANCA)-associated vasculitis is a rare but life-threatening autoimmune disease which can have multi-organ involvement. It is usually characterized by inflammation and damage to small blood vessels eventually causing involved tissue/organ damage. Common presenting symptoms include arthralgias, myalgias, anorexia, fever, and malaise but can also have organ involvement causing renal failure in the form of pauci-immune glomerulonephritis and acute respiratory failure in the form of pulmonary alveolar hemorrhage [1-3]. Although aggravating factors remain unclear in idiopathic ANCA vasculitis, there are some drugs which have been associated with drug-induced ANCA vasculitis. In this case report, we intend to describe this rare manifestation of drug-induced vasculitis from a very commonly used antihypertensive namely hydralazine. While it is generally considered to be a safe and effective treatment, in rare cases it can cause a serious side effect known as hydralazine-induced vasculitis with multi-organ involvement with the most common being renal involvement.

## Case presentation

The patient is a 67-year-old female with a past medical history of chronic obstructive pulmonary disease (COPD), congestive heart failure (CHF), hypertension, hyperlipidemia, left renal artery stenosis status post stenting two months prior to the first visit with nephrology, chronic kidney disease (CKD) stage IIIA with the recent progression of CKD. Prior to this initial office visit with Nephrology, the patient was admitted to a local community hospital for CHF exacerbation. She was discharged to home with 40 mg Lasix daily because she was still in volume overload. The usual dose before this admission was 20 mg daily. The patient was found to have some chronic kidney disease going back to 2010 when serum creatinine was 1.2 but fluctuating intermittently. At the time of discharge from the hospital, serum creatinine was 1.96. Ultrasound kidney done around the time of presentation showed the right kidney measuring 9.7 cm and the left kidney measuring 9.9 cm in length with normal bilateral renal cortical thickness but has renal parenchymal echogenicity. No calculi or solid renal mass was seen. Small left renal cortical cyst was present. The patient denied any chronic use of non-steroidal anti-inflammatory drugs (NSAIDs), frothy urine, flank pain, or dysuria with her kidney dysfunction. The patient continued to have shortness of breath which has not changed for the last few days but she remained short of breath and cannot even complete a sentence without getting short of breath. Also reported decreased appetite and weight loss over the period of last one year but denied any chest pain, fever, nausea, vomiting, diarrhea, abdominal pain, and dysuria.

**Figure 1:**
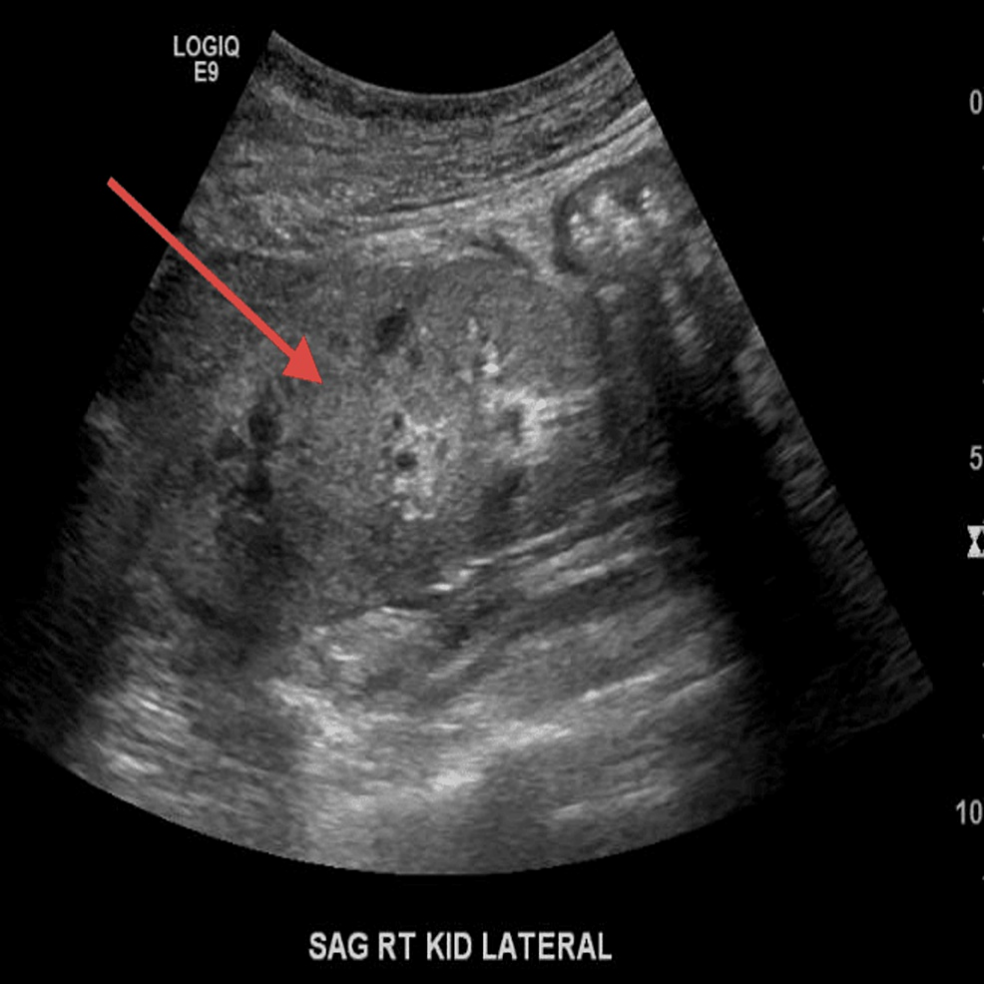
Ultrasound image of the right kidney Ultrasound of the right kidney showing echogenic parenchyma (red arrow).

**Figure 2:**
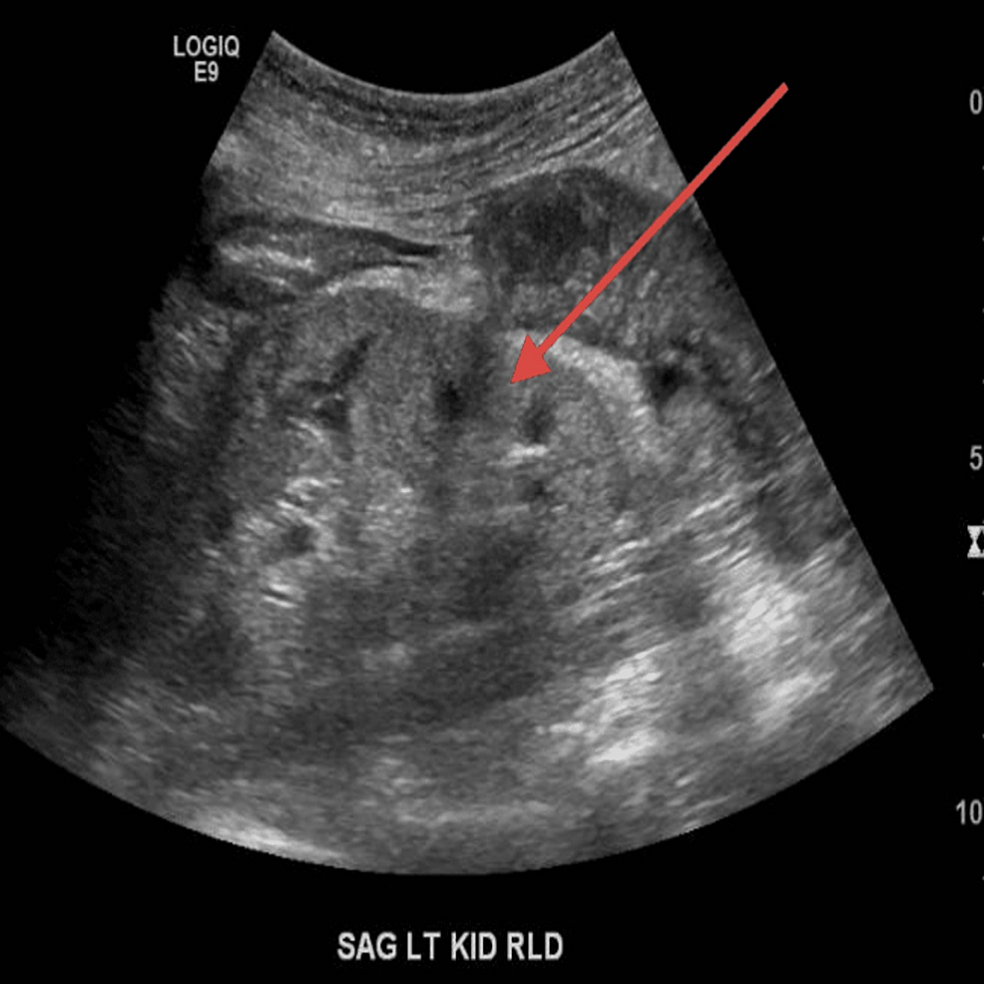
Ultrasound image of the left kidney Ultrasound of the left kidney showing echogenic parenchyma (red arrow).

**Figure 3:**
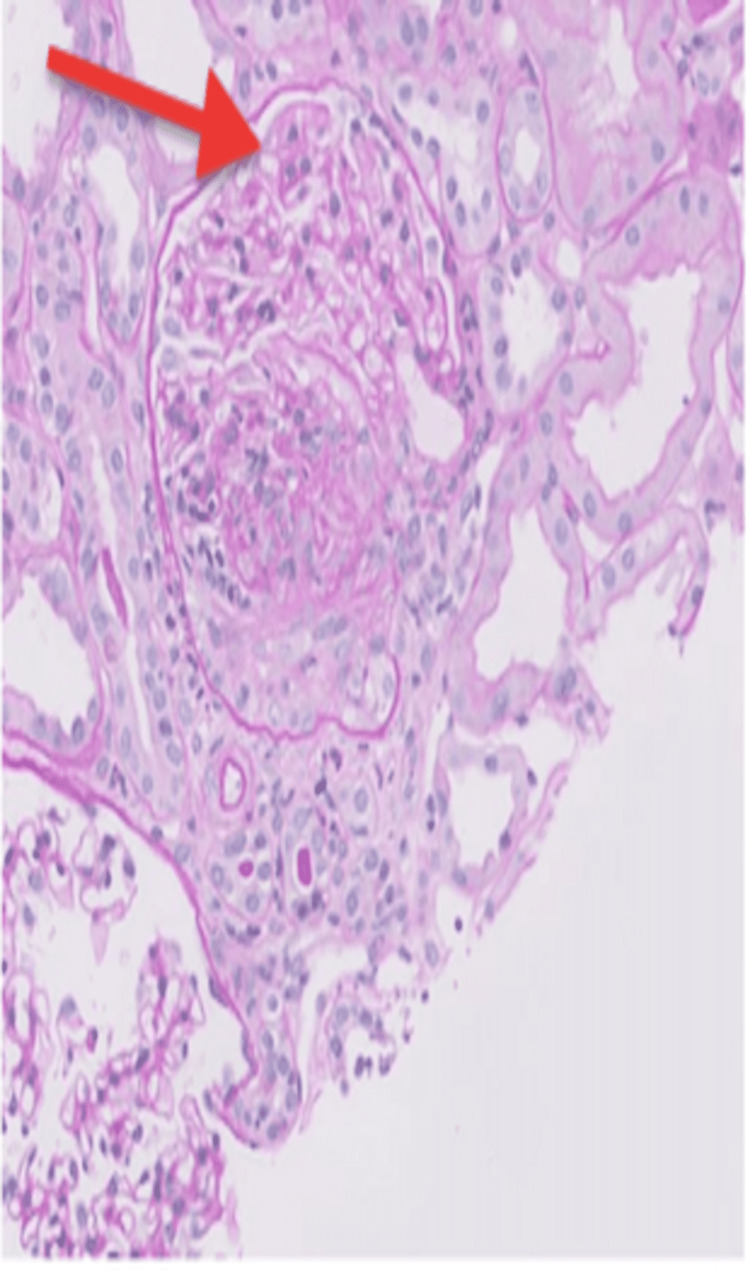
Renal biopsy pathology (H&E slide) Very focal crescentic necrotizing glomerulonephritis (red arrow).

On examination, the temperature was 97 degrees Fahrenheit, blood pressure 150/92 mm Hg, heart rate 80 beats per minute, respiratory rate 20 breaths per minute, and oxygen saturation 97% while the patient was breathing on room air. The body-mass index (the weight in kilograms divided by the square of the height in meters) was 17.1. A cardiopulmonary exam revealed a normal rate and rhythm without adventitious heart sounds. The mucous membranes were moist. Trace edema was noted in the bilateral lower extremities. Bilateral lung exam was clear to auscultation. The remainder of the physical examinations was unremarkable. Presentation labs were notable for serum sodium 134 mmol/l, potassium 4.3 mmol/l, chloride 99 meq/l, CO2 26 meq/l, blood urea nitrogen 34 mg/dl, creatinine 1.96 mg/dl, calcium 9.1, and albumin 3.7. Urine analysis was significant for microscopic hematuria 6-10 RBC, no proteinuria. At this visit, an immunological workup was sent to evaluate the presence of hematuria and unexplained worsening of kidney function. The patient was also referred to Urology for cystoscopy for ruling out bladder cancer in a setting of hematuria with a history of smoking and recent weight loss. A follow-up visit was planned in three weeks with repeat renal function, US renal artery doppler, and immunological workup. Repeat renal function revealed a worsening in creatinine to 3.1 mg/dl from 1.9 mg/dl four weeks back. A repeat renal artery doppler was done to check the patency of the renal stent placed in the left renal artery and came back normal. Ultrasound of the kidney revealed the right kidney measures 9.3 cm with diffusely increased echogenicity (Figure 1) relative to the liver. The left kidney measures 9.4 cm with diffusely increased echogenicity (Figure 2). Immunological workup came with elevated ANCA titers at 1:10240 (Reference range <1:20) with p-ANCA titers at >1:640 (Reference range <1:20), Myeloperoxidase AB level 156 AU/ml (Reference range 0-19 AU/ml), PR3 AB mildly elevated at 32 AU/ml (Reference range 0-19 AU/ml), positive ANA 1:2560 (Reference range <1:80), positive DS-DNA 1:20 (Reference range <1:10), low C3 77 mg/dl (Reference range 90-180 mg/dl), normal c4 23 mg/dl (Reference range 18-45 mg/dl). This prompted admission to the inpatient medicine floor for solumedrol induction and renal biopsy. Renal biopsy revealed very focal crescentic (red arrow) glomerulonephritis, an increased number of occlusive red blood cell casts with acute tubular necrosis. Mild interstitial fibrosis of <20% was seen (Figure 3). Mild staining for IgA and IgG on immunofluorescence depicted pauci-immune nature of immune deposit. As hydralazine has been reported to cause both drug-induced lupus and drug-induced ANCA vasculitis and renal biopsy showed crescentic and pauci-immune glomerulonephritis, a diagnosis of hydralazine-induced ANCA vasculitis was made. After the withdrawal of hydralazine, the patient was treated with high-dose prednisone (1 mg/kg) and rituximab infusion but unfortunately, she went into sepsis because of her frail status and a decision was taken to withdraw immunosuppression. Her chronic kidney disease progressed and eventually started on peritoneal dialysis.

## Discussion

Antineutrophil cytoplasmic antibody (ANCA)-associated vasculitides (AAVs) are a group of diseases (granulomatosis with polyangiitis, eosinophilic granulomatosis with polyangiitis, and microscopic polyangiitis), characterized by destruction and inflammation of small vessels. The clinical signs vary and affect several organs, such as the kidney, stomach, intestine, and lungs. Skin lesions, such as purpura and urticaria, result when blood from small vessels leaks under the skin. AAVs occur when neutrophils attack small and medium vessels of the body.

AAVs are associated with significant morbidity and mortality, with almost all patients requiring aggressive immunosuppression. Without treatment, the prognosis is poor [1]. Based upon the clinical presentation and the predominant organ involvement, AAV cases are classified as Wegener’s granulomatosis (WG), microscopic polyangiitis (MPA), Churg-Strauss syndrome (CSS), and Renal Limited Vasculitis (RLV).

A recent development in the field of vasculitis is the increasing recognition that drugs from almost every pharmacological class are implicated in causing vasculitis in sporadic cases and the demonstration of ANCA has revealed an interesting subset of drug-induced vasculitis (DIV). Certain medications may induce forms of vasculitis associated with ANCA (Table 1). Most patients reported with drug-induced ANCA-associated vasculitis have MPO-ANCA, frequently in very high titers [4]. Here we discuss a case of a patient presenting with hydralazine-induced vasculitis.

**Table 1:** Medications associated with drug-induced vasculitis

Medications associated with drug-induced vasculitis
Antibiotics
Cefotaxime
Minocycline
Anti-thyroid drugs
Benzylthiouracil
Carbimazole
Methimazole
Propylthiouracil

Anti-tumor necrosis factor-a agents
Adalimumab
Etanercept
Infliximab

Psychoactive agents
Clozapine
Thioridazine

Miscellaneous drugs
Allopurinol
D-Penicillamine
Hydralazine
Levamisole
Phenytoin

Hydralazine is a medication that is commonly used to treat hypertension or high blood pressure. While it is generally considered to be a safe and effective treatment, in rare cases it can cause a serious side effect known as hydralazine-induced vasculitis.

Vasculitis is a condition in which the blood vessels become inflamed, leading to damage and narrowing of the vessels. This can restrict blood flow and oxygen to the body's tissues and organs, potentially causing serious complications.

Symptoms of hydralazine-induced vasculitis can include fever, joint pain, skin rash, and other signs of inflammation. The condition can also cause changes in skin color and temperature, as well as numbness or tingling in the fingers and toes. In more severe cases, vasculitis can lead to organ damage, such as damage to the heart, lungs, or kidneys.

While the exact cause of hydralazine-induced vasculitis is not known, it is thought to be related to an immune system reaction to the medication. There might also be a genetic predisposition for drug-induced vasculitis as shown with the use of minocycline [5].

Hydralazine-induced ANCA vasculitis is more often found to have renal involvement in the form of pauci-immune glomerulonephritis and is usually associated with antibodies to double-stranded DNA, very high titers of MPO-ANCA as well as anti-histone antibodies and hypocomplementemia. All of these lab biomarkers and the temporal relationship between the start of the medication and the onset of symptoms are helpful diagnostic tools to diagnose this rare condition. Treatment usually involves stopping the medication as soon as the suspicion of vasculitis arises [4,6,7]. In most cases with renal involvement and severe symptoms, immunosuppressive therapy is required including glucocorticoids, rituximab, cyclophosphamide, and/or mycophenolate with or without plasmapheresis [8].

It's important to note that hydralazine-induced vasculitis is a rare side effect of the medication, and most people who take it do not experience this complication.

## Conclusions

In conclusion, hydralazine is a medication used to treat hypertension, it can cause a rare side effect known as hydralazine-induced vasculitis - a condition where the blood vessels become inflamed, leading to damage and narrowing of the vessels. Symptoms include fever, joint pain, skin rash, other signs of inflammation, and organ damage. It is important to discontinue the medications as soon as vasculitis is suspected and should be confirmed with laboratory biomarkers. Some patients might need immunosuppressive therapy if symptoms are severe or if there is renal involvement.

## References

[1] Kitching AR, Anders HJ, Basu N (2020). ANCA-associated vasculitis. Nat Rev Dis Primers.

[2] Hoffman GS, Kerr GS, Leavitt RY (1992). Wegener granulomatosis: an analysis of 158 patients. Ann Intern Med.

[3] Falk RJ, Hogan S, Carey TS, Jennette JC (1990). Clinical course of anti-neutrophil cytoplasmic autoantibody-associated glomerulonephritis and systemic vasculitis. The Glomerular Disease Collaborative Network. Ann Intern Med.

[4] Choi HK, Merkel PA, Walker AM, Niles JL (2000). Drug-associated antineutrophil cytoplasmic antibody-positive vasculitis: prevalence among patients with high titers of antimyeloperoxidase antibodies. Arthritis Rheum.

[5] Kawahara H, Nakashima A, Zoshima T, Kawano M (2020). Contribution of HLA-DRB1 * 09: 01 allele to development of minocycline induced antineutrophil cytoplasmic antibody (ANCA)-associated cutaneous vasculitis: report of two cases. Mod Rheumatol Case Rep.

[6] Short AK, Lockwood CM (1995). Antigen specificity in hydralazine associated ANCA positive systemic vasculitis. QJM.

[7] Al-Abdouh A, Siyal AM, Seid H, Bekele A, Garcia P (2020). Hydralazine-induced antineutrophil cytoplasmic antibody-associated vasculitis with pulmonary-renal syndrome: a case report. J Med Case Rep.

[8] Santoriello D, Bomback AS, Kudose S (2021). Anti-neutrophil cytoplasmic antibody associated glomerulonephritis complicating treatment with hydralazine. Kidney Int.

